# Association Between Genetically Predicted Memory and Self-Reported Foreign Language Proficiency

**DOI:** 10.3390/genes16050589

**Published:** 2025-05-17

**Authors:** Meruert B. Yerdenova, Gaukhar K. Datkhabayeva, Manzura K. Zholdassova, Altyngul T. Kamzanova, Zukhra M. Sadvakassova, Amal Bouzid, Poorna Manasa Bhamidimarri, Rifat Hamoudi, Ekaterina A. Semenova, Andrey K. Larin, Nikolay A. Kulemin, Edward V. Generozov, Tim Rees, Almira M. Kustubayeva, Ildus I. Ahmetov

**Affiliations:** 1 Department of General and Applied Psychology, Al-Farabi Kazakh National University, Almaty 050040, Kazakhstan; 2 Department of Biophysics, Biomedicine and Neuroscience, Center for Cognitive Neuroscience, Al-Farabi Kazakh National University, Almaty 050040, Kazakhstan; gaukhar.datkhabayeva@kaznu.edu.kz (G.K.D.);; 3 Brain Institute, Al-Farabi Kazakh National University, Almaty 050040, Kazakhstan; 4 Sharjah Institute for Medical Research, College of Medicine, University of Sharjah, Sharjah 27272, United Arab Emiratesr.hamoudi@ucl.ac.uk (R.H.); 5 Division of Surgery and Interventional Science, University College London, London NW3 2PF, UK; 6 Department of Molecular Biology and Genetics, Lopukhin Federal Research and Clinical Center of Physical-Chemical Medicine of Federal Medical Biological Agency, 119435 Moscow, Russiagenerozov@gmail.com (E.V.G.); 7 Research Institute of Physical Culture and Sport, Volga Region State University of Physical Culture, Sport and Tourism, 420138 Kazan, Russia; 8 Department of Rehabilitation and Sport Science, Faculty of Health and Social Sciences, Bournemouth University, Bournemouth BH12 5BB, UK; trees@bournemouth.ac.uk; 9 Laboratory of Genetics of Aging and Longevity, Kazan State Medical University, 420012 Kazan, Russia; 10 Research Institute for Sport and Exercise Sciences, Liverpool John Moores University, Liverpool L3 5AF, UK

**Keywords:** DNA, genotype, genetic markers, intelligence, linguistic immersion, language abilities, cognitive development, behavior genetics, cognitive abilities

## Abstract

Background: Foreign language proficiency is a complex trait that reflects an individual’s ability to effectively understand and use a non-native language, shaped by both genetic and environmental factors. The aim of this study was to establish the relationship between genetically determined memory capacity and self-reported foreign language proficiency in 129 children (63 males, 66 females, age 14.2 ± 3.9) and 128 adults (90 males, 38 females, age 29.8 ± 8.2). Methods: Seven single nucleotide polymorphisms (SNPs) previously linked with memory function were used in a polygenic analysis (*CAMTA1* rs4908449, *CLSTN2* rs6439886, *COMT* rs4680, *CPEB3* rs11186856, *SCN1A* rs10930201, *SNAP25* rs3746544, and *WWC1* rs17070145). Self-reported foreign language proficiency was evaluated using a single-item question. Children’s level of immersion in foreign languages was divided into three categories: linguistic school, non-linguistic school with extra foreign language courses, and non-linguistic school without additional foreign language courses. Results: We found that genetically predicted memory capacity (i.e., number of memory-increasing alleles) was positively associated with self-reported foreign language proficiency in children (*p* = 0.0078 adjusted for age, sex, ethnicity, verbal IQ, and level of immersion in foreign languages). When combined, genetically predicted memory capacity, age, sex, ethnicity, verbal IQ, and level of immersion in foreign languages explained 31.5% (*p* < 0.0001) of the variance in children’s self-reported foreign language proficiency. The association between genetically predicted memory capacity and self-reported foreign language proficiency was replicated in adults (*p* = 0.0158 adjusted for age, sex, and ethnicity). Conclusions: Foreign language proficiency may partly depend on the presence of a high number of memory-increasing alleles in both children and adults.

## 1. Introduction

Language proficiency is a complex, diverse, cognitive trait that is crucial for both personality and societal development. Encoding, storing, and retrieving a large quantity of information, such as vocabulary and grammatical rules, is necessary for successful language acquisition [1]. Over the past decades, a wide range of environmental, cognitive, and socio-demographic factors influencing language acquisition have been identified, including age, gender, language immersion, culture, socioeconomic status, and intellectual capacity [2]. Studies have shown a consistent relationship between multilingualism and intellectual capacity: in general, the more languages a person learns, the higher their IQ [3]. It is reasonable to assume that people with higher intellectual capacity may possess enhanced cognitive abilities that enable them to process, understand, and apply multiple languages more quickly, because language learning is more complex than simply memorizing vocabulary and grammar rules.

As Dörnyei [2] noted, in *The Psychology of the Language Learner*, the process of second language acquisition is profoundly shaped by individual differences—that is, one often observes diverse outcomes across learners despite similar instructional environments. Of these individual differences, personality traits such as extraversion and openness to experience may enhance engagement with language tasks [2,4], as does “strategic behavior”—such as systematic vocabulary practice or self-monitoring [2,5]. Even language anxiety, when experienced in moderation, can encourage learners to invest greater effort in preparation [2,6]. However, the most influential predictors are motivation, age of onset, and, of particular relevance to the present study, language aptitude [2,7,8,9,10].

Although the role of practice within second language acquisition is widely acknowledged [11], genetics also plays a crucial role in shaping language skills [12]. Indeed, intrinsic biological heterogeneity among people has long been acknowledged, with cognitive talents (including language skills) assumed to be among the many phenotypic qualities displaying this variation [13].

The connection between memory function and fluency in a foreign language has been of interest to educational psychologists and neurobiologists. Theoretical predictions suggest that genetics may predict not just memory capacity but also second language acquisition ability [14]. Genetic variables impacting memory function have also been explored [15]. The idea that genes have a role in memory is not new; in fact, it goes back to the middle of the 20th century, when researchers first began looking at how genes affect human cognition [16]. Twin studies have provided strong evidence that individual variations in memory function are mostly caused by genetic variation [17]. Different facets of memory functioning have been linked to genes, including *CAMTA1*, *CLSTN2*, *COMT*, *CPEB3*, and *KIBRA* (*WWC1*), among others [18].

Early studies looking at the relationship between memory, heredity, and learning a foreign language mostly focused on dyslexia and other learning difficulties [19]. Language acquisition is often challenging for people with dyslexia, something which has been linked in part to hereditary memory-affecting variables [20]. By the 21st century, however, improvements in genetic analysis methods made it possible for researchers to examine these connections on a genome-wide scale [21]. Importantly, improvements in genetic methodologies now allow for the construction of polygenic scores—indices reflecting the cumulative effect of multiple alleles—providing a powerful tool to examine the genetic contributions to complex traits such as language learning.

One might hypothesize that if some genetic markers predict memory function, they will also underlie a person’s innate predisposition for second language acquisition. Indeed, research has revealed the pivotal role of phonological working memory as a predictor of successful foreign language acquisition, particularly during the early stages of learning [22]. Specifically, the quality of pronunciation in an auditory pseudoword repetition task and the accuracy of spelling in a visual delayed copying task with pseudowords were found to correlate with elementary foreign language learning [22]. Given this background, the present study aimed to explore whether a greater number of memory-enhancing alleles predicts higher foreign language proficiency across the lifespan. We addressed this question in two independent cohorts of children and adults, while carefully controlling for important confounding factors such as age, sex, ethnicity, verbal IQ, and language immersion environment.

The specific objectives of the study were:

- To investigate the association between a polygenic memory score and self-reported foreign language proficiency in children.
- To replicate this association in an independent adult cohort.
- To assess the combined contribution of genetically predicted memory capacity, age, sex, ethnicity, verbal IQ, and level of immersion in foreign languages to children’s self-reported foreign language proficiency.

To test these hypotheses, we analyzed seven SNPs (*CAMTA1* rs4908449, *CLSTN2* rs6439886, *COMT* rs4680, *CPEB3* rs11186856, *SCN1A* rs10930201, *SNAP25* rs3746544, and *WWC1* rs17070145) previously linked to memory performance in candidate gene or genome-wide association studies. A polygenic approach was chosen to capture the cumulative effect of small genetic contributions [23,24]. Self-reported proficiency was used due to its strong correlation with objective language measures across multiple studies [25,26,27], and immersion levels were categorized to account for environmental influences. By integrating genetic, cognitive, and environmental data, this study offers a novel perspective on the biological underpinnings of language learning, paving the way for personalized educational strategies.

## 2. Materials and Methods

### 2.1. The Ethics Statement

The Ethics Committees of the Al-Farabi Kazakh National University (Approval numbers: IRB-A172 and IRB-A267) and the Federal Research and Clinical Center of Physical-Chemical Medicine of the Federal Medical and Biological Agency of Russia (Approval number 2017/04) approved the protocols for the research. Informed consent was obtained from all participants (and parents or legal guardians, where appropriate) involved in the study. The study was conducted according to the guidelines of the Declaration of Helsinki and Strengthening The Reporting of Genetic Association Studies (STREGA): An extension of the Strengthening the Reporting of Observational Studies in Epidemiology (STROBE) statement recommendations.

### 2.2. Participants

The first cohort comprised 129 healthy children (63 males, 66 females; 111 Kazakhs, 18 Russians; age 14.2 ± 3.9; age range 7–21) from Kazakhstan. The children attended different schools, where the first language was either Kazakh (*n* = 63) or Russian (*n* = 66). The pupils either went to a school with an in-depth study of foreign languages (linguistic), a non-linguistic school that offered extra courses in foreign languages, or a non-linguistic school that did not provide extra courses in foreign languages. Because it was expected that the amount of immersion in foreign languages may have an effect on language competency, the design of the research took into consideration the distribution of pupils throughout these different kinds of schools.

The second cohort comprised 128 healthy adults (90 males, 38 females; 107 Russians, 21 Ukrainians and Belarusians; age 29.8 ± 8.2; age range 18–54) from Russia. Russians, Belarusians, and Ukrainians belong to the East Slavic group of Eastern Europeans. This cohort was previously described in detail [28], and a portion of that cohort had agreed to answer questions about foreign language proficiency.

### 2.3. Psychometric Methods

To determine engagement with foreign languages and foreign language proficiency, participants (in conjunction with their parents, where appropriate) were asked to respond to questions regarding (a) what second languages they spoke (open-ended), (b) their level of immersion in foreign languages (for children only: linguistic school, non-linguistic school with extra foreign language courses, and non-linguistic school without additional foreign language courses; coded as 3, 2, 1, respectively), and (c) their self-reported level of foreign language proficiency (rated as beginner, elementary, intermediate, or advanced—coded as 1, 2, 3, or 4, respectively). For most participants, their second (foreign) language was English (128 children and 124 adults); for four participants, their second language was German; and for one participant, their second language was French.

Intelligence was measured in children only using the Wechsler test, which includes 11 separate component sub-tests across six verbal and five non-verbal aspects. The study used two versions of the Wechsler test for two age categories of participants:

(a) 7–15 years old: Wechsler Intelligence Scale for Children (WISC) test—for testing children and adolescents [29]. The children’s version of this test was adapted and standardized for Russian speakers by A. Yu. Panasyuk [30].
(b) 16–21 years old: Wechsler Adult Intelligence Scale (WAIS) test—designed to test adults [31]. This version was adapted and standardized for Russian speakers by A. Yu. Panasyuk, and supplemented and corrected by Yu. I. Filimonenko and V. I. Timofeev at the State Enterprise “Imaton”, St. Petersburg [32].

Both intelligence test versions were translated into the Kazakh language for those children for whom the Kazakh language was their first language. Cronbach’s α internal consistency reliability for the intelligence tests within the present samples is shown in Supplementary Table S1.

### 2.4. Genetic Analysis

In this study, seven key genetic markers that are connected with memory capacity were selected and genotyped in the studied samples (Table 1). Genetic markers were selected based on reproducibility of results, sample size of each study, and methodology (some markers were discovered in the genome-wide association studies, such as *CLSTN2* rs6439886, *SCN1A* rs10930201, and *WWC1* rs17070145).

### 2.5. Genotyping of Children’s DNA Samples

Samples were collected using a non-invasive method of sampling the epithelium of cells from the oral cavity of the 129 participants. DNA was extracted from the buccal swab samples using a QIAamp DNA Mini kit (Cat No. 51306; Qiagen, Hilden, Germany) according to the manufacturer’s instructions. Samples were processed as previously described [47]. Genomic DNA quantity and quality were assessed using a Nanodrop2000 spectrophotometer (Thermo Scientific, Waltham, MA, USA). In silico primer design was performed to cover the seven selected SNPs. The primer sequences used for genotyping are listed in Supplementary Table S2.

First, the primers were evaluated using control DNA samples, and the expected PCR product size was validated using agarose gel electrophoresis. Next, the primers were tagged with Fluidigm-specific tag sequences CS1: ACACTGACGACATGGTTCTACA for the forward primer, and CS2: TACGGTAGCAGAGACTTGGTCT for the reverse primer. The libraries for DNA sequencing using the Fluidigm Access Array microfluidic chip were generated as previously described [48]. Samples were pooled and sequenced using the Ion 520™ Chip on the Ion S5 XL Semiconductor sequencer following the manufacturer’s instructions (Thermo Fischer, Waltham, MA, USA). The genomic data were treated using an in-house bioinformatics pipeline, including alignment to the reference genome GRCh37/hg19, quality control assessment, SNP calling, and variant annotation, as previously described [49]. SNP genotyping of the seven studied markers was collected for all samples. The functional annotation of the variants was performed using the Ensembl Variant Effect Predictor tool [50].

### 2.6. Genotyping of Adults’ DNA Samples

Molecular genetic analysis was performed with DNA samples obtained from leukocytes (venous blood). Four ml of venous blood was collected in tubes containing EDTA (Vacuette EDTA tubes, Greiner Bio-One, Kremsmünster, Austria). DNA extraction and purification were performed using a commercial kit according to the manufacturer’s instructions (Technoclon, Moscow, Russia). HumanOmniExpressBeadChips (Illumina Inc., San Diego, CA, USA) were used to genotype seven polymorphisms, as previously described [51].

### 2.7. Statistical Analyses

All statistical analyses were performed using GraphPad InStat v. 3.05 (GraphPad Software, Inc., San Diego, CA, USA). A polygenic memory score was calculated by summing the number of memory-increasing alleles across the selected SNPs (range: 0–14 for seven SNPs, 0–12 for six SNPs, and 0–10 for five SNPs), assuming an additive genetic model (0, 1, or 2 favorable alleles per SNP). Each SNP was previously associated with memory performance in the literature, and the favorable allele for each SNP was defined based on prior evidence of its positive association with memory. This total score was then included as a continuous independent variable in the multiple linear regression models predicting self-reported foreign language proficiency. Standard multiple linear regression (ordinary least squares method) was used to examine the association between self-reported foreign language proficiency (continuous outcome variable) and genetically predicted memory capacity, as well as other covariates. For children, the predictor variables included sex, age, ethnicity, verbal IQ, and level of immersion in foreign languages. For adults, the predictor variables included sex, age, and ethnicity. The coefficient of determination (R^2^) was used to quantify the proportion of variance in foreign language proficiency explained by the combined predictors. Hardy–Weinberg equilibrium was assessed for each SNP. All data are reported as mean (standard deviation), and statistical significance was set at *p* < 0.05.

## 3. Results

The genotype distribution and allelic frequencies of the seven SNPs linked to memory function in the two cohorts are shown in Table 2. In children, two SNPs (*COMT* rs4680 and *SCN1A* rs10930201), and in adults, one SNP (*CAMTA1* rs4908449), did not meet Hardy–Weinberg equilibrium criteria (Table 2).

A comparison of memory-increasing allele frequencies for five polymorphisms (*CAMTA1* rs4908449, *CLSTN2* rs6439886, *CPEB3* rs11186856, *SNAP25* rs3746544, and *WWC1* rs17070145) among Kazakh children, Russian children living in Kazakhstan, Slavic adults living in Russia, and two 1000 Genomes Project populations—East Asians and Europeans [52]—revealed that Kazakh children often display intermediate frequencies between East Asians and Europeans, reflecting their admixed ancestry (Supplementary Table S3). Our analysis further revealed that Russian children residing in Kazakhstan share a common Slavic/European ancestry with Slavic adults, as demonstrated by their closely aligned allele frequencies for *CLSTN2* rs6439886, *SNAP25* rs3746544, and *WWC1* rs17070145. However, a subtle overall shift in Russian children toward Kazakh children, particularly in other polymorphisms, likely reflects localized genetic influences stemming from admixture or environmental factors in Kazakhstan (Supplementary Table S3).

The number of memory-increasing (favorable) alleles for seven SNPs (minimum—0, maximum—14) ranged from 2 to 10 for children, and from 2 to 11 for adults. Sex had no significant effect on any of the tested variables of the children, including verbal IQ (*p* = 0.402), self-reported foreign language proficiency (*p* = 0.3899), level of immersion in foreign languages (*p* = 0.756), and number of memory-increasing alleles (*p* = 0.312) (Supplementary Table S4). In addition, ethnicity (*p* = 0.9948) had no effect on self-reported foreign language proficiency. Furthermore, there were no significant differences in the number of memory-increasing alleles between Kazakhs and Russians living in Kazakhstan. We therefore felt justified to combine all participants into one group for further analyses (Supplementary Table S5). In children, we found that genetically predicted memory capacity (number of memory-increasing alleles based on seven SNPs) was positively associated with self-reported foreign language proficiency (β = 0.117, *p* = 0.0078, adjusted for age, sex, ethnicity, verbal IQ, and level of immersion in foreign languages). This association remained significant when only Kazakh children were analyzed (β = 0.103, *p* = 0.0306; adjusted for age, sex, verbal IQ, and level of immersion in foreign languages). Furthermore, age (β = 0.091; *p* < 0.0001), level of immersion in foreign languages (β = 0.294; *p* = 0.0035), and verbal IQ (β = 0.020; *p* = 0.0004) were also positively associated with children’s self-reported foreign language proficiency. When combined, genetically predicted memory capacity, age, sex, ethnicity, verbal IQ, and level of immersion in foreign languages explained 31.5% (*p* < 0.0001) of the variance in children’s self-reported foreign language proficiency in the seven SNPs model. After excluding two SNPs (*COMT* rs4680 and *SCN1A* rs10930201) that did not meet Hardy–Weinberg equilibrium criteria, a memory polygenic score based on the remaining five SNPs remained significantly associated with children’s self-reported foreign language proficiency (β = 0.147, *p* = 0.0091), after adjusting for age, sex, ethnicity, verbal IQ, and level of immersion. Age (β = 0.096; *p* < 0.0001), immersion (β = 0.283; *p* = 0.0048), and verbal IQ (β = 0.02; *p* = 0.0003) also showed positive associations. Together, these factors explained 31.3% (*p* < 0.0001) of the variance in language proficiency in the five SNPs model.

In adults, age (*p* = 0.1803), sex (*p* = 0.1492), and ethnicity (*p* = 0.7986) had no effect on self-reported foreign language proficiency (Supplementary Table S4). Furthermore, there were no significant differences in the number of memory-increasing alleles between Russians and the other two East Slavic ethnic groups (Belarusians and Ukrainians). We therefore felt justified to combine all participants into one group for further analyses (Supplementary Table S5). The positive association between genetically predicted memory capacity (based on seven SNPs) and self-reported foreign language proficiency was replicated in adults (β = 0.103, *p* = 0.0158 adjusted for age, sex, and ethnicity). This association remained significant after excluding one SNP (*CAMTA1* rs4908449) that did not meet Hardy–Weinberg equilibrium criteria (β = 0.100, *p* = 0.0371; adjusted for age, sex, and ethnicity).

## 4. Discussion

To the best of our knowledge, this is the first study to show that genetically determined memory capacity is positively associated with self-reported foreign language proficiency. Alongside this key finding, we demonstrated that age, level of immersion in foreign languages, and verbal IQ were also positively associated with self-reported foreign language proficiency. Of particular note, we were able to replicate our findings in children with a separate sample of adults.

Our study was based on a body of past research showing that multiple cognitive skills, including working memory, are important for effective language learning [22]. Seven genetic variants used in our research have previously been linked to memory and other cognitive-related traits, such as spatial ability, intelligence, and educational attainment. These variants are located in genes (*CAMTA1*, *CLSTN2*, *COMT*, *CPEB3*, *SCN1A*, *SNAP25*, and *WWC1*) responsible for neurological processes that underlie memory and cognitive function, including synaptic plasticity, neurogenesis, and neurotransmission.

The *CAMTA1* gene encodes calmodulin-binding transcription activator 1, which interfaces with the calcium–calmodulin system of the cell to alter gene expression patterns [33]. Carriers of the *CAMTA1* rs4908449 T allele have been shown to demonstrate better performance in an episodic recall memory test [33]. The *CLSTN2* gene encodes calsyntenin 2 and is involved in positive regulation of synapse assembly and positive regulation of synaptic transmission [35]. Carriers of the *CLSTN2* rs6439886 G allele have better episodic memory [34,35,36]. The *COMT* gene encodes catechol-O-methyltransferase, which catalyzes the transfer of a methyl group from S-adenosylmethionine to catecholamines, including the neurotransmitters dopamine, epinephrine, and norepinephrine. The *COMT* rs4680 A allele has been reported to be associated with better verbal working memory [37], language ability [39], spatial working memory [38], and visuospatial and social working memory [40]. The *CPEB3* gene encodes cytoplasmic polyadenylation element-binding protein 3, which is crucial for synaptic plasticity and memory in model organisms [40]. The *CPEB3* rs11186856 A allele is associated with episodic memory [41]. The *SCN1A* gene encodes the sodium voltage-gated channel α subunit 1 and regulates the release of neurotransmitters in neurons. The *SCN1A* rs10930201 A allele has been linked with short-term memory [42]. The *SNAP25* gene encodes synaptosome-associated protein 25, which plays an important role in the synaptic function of specific neuronal systems. The *SNAP25* rs3746544 G allele has been reported to be associated with better brain functional connectivity density and working memory [43]. The *WWC1* (also known as *KIBRA*) gene encodes WW and C2 domain-containing 1 protein, which, together with its binding partners (dendrin, synaptopodin, dynein-complex, and others), plays an important role in synaptic plasticity [34]. The *WWC1* gene rs17070145 T allele has been linked with better episodic and working memory [34,35,36,44,46] and verbal memory [45].

Against the backdrop of this study’s novel findings, there are some limitations that should be acknowledged. First, our cohort of children was heterogeneous with respect to age, ethnicity, and level of immersion in foreign languages. We therefore adjusted our findings for these and other (sex and verbal IQ) covariates. Second, to test foreign language proficiency, we used a self-reported phenotype (a survey question), which was swift to administer, but we acknowledge the availability of various objective assessments of foreign language proficiency (e.g., TOEFL, IELTS, etc.). Finally, our study is limited to the seven common polymorphisms which were primarily selected because of previously reported associations with memory capacity. It is likely, however, that future research will show that many additional common polymorphisms, and probably rare mutations as well, are associated with memory capacity and foreign language proficiency.

Overall, our research provides strong support for the idea that memory function and language aptitude are genetically linked. Indeed, the present study suggests that self-reported foreign language proficiency may partly depend on the presence of a high number of memory-increasing alleles in both children and adults. The process behind genetically predicted memory capacity and self-reported foreign language proficiency needs to be further investigated in order to develop individualized teaching methods and interventions targeted at enhancing language learning results. This study also highlights that researchers examining cognitive variables and educational policy-makers would be well-advised to consider taking into account the influence of genetic variables, especially with regard to memory and language development.

## Figures and Tables

**Table 1:** List of selected genetic markers associated with memory.

Gene	Polymorphism	Alleles	Favorable Allele	References
*CAMTA1*	rs4908449	T/C	T	[33]
*CLSTN2*	rs6439886	A/G	G	[34,35,36]
*COMT*	rs4680	G/A	A	[37,38,39,40]
*CPEB3*	rs11186856	A/G	A	[41]
*SCN1A*	rs10930201	A/C	A	[42]
*SNAP25*	rs3746544	G/T	G	[43]
*WWC1*	rs17070145	C/T	T	[34,35,36,44,45,46]

**Table 2:** Genotype and allele frequencies of 7 memory-related SNPs in children (*n* = 129) and adults (*n* = 128).

Polymorphism	Genotype 1	Genotype 2	Genotype 3	Hardy–Weinberg Equilibrium		Memory-Increasing Allele Frequency, %
				χ^2^	*p*	
Children						
*CAMTA1* rs4908449	TT (20)	TC (49)	CC (60)	3.28	0.070	T (34.5)
*CLSTN2* rs6439886	GG (0)	AG (19)	AA (110)	0.82	0.366	G (7.4)
*COMT* rs4680	AA (18)	GA (19)	GG (92)	40.59	<0.0001	A (21.3)
*CPEB3* rs11186856	AA (107)	AG (19)	GG (3)	3.24	0.072	A (90.3)
*SCN1A* rs10930201	AA (39)	AC (1)	CC (89)	124.33	<0.0001	A (30.6)
*SNAP25* rs3746544	GG (11)	GT (67)	TT (51)	2.87	0.090	G (34.5)
*WWC1* rs17070145	TT (42)	CT (67)	CC (20)	0.63	0.427	T (58.5)
Adults						
*CAMTA1* rs4908449	TT (25)	TC (46)	CC (57)	6.97	0.008	T (37.5)
*CLSTN2* rs6439886	GG (2)	AG (30)	AA (96)	0.04	0.843	G (13.3)
*COMT* rs4680	AA (32)	GA (70)	GG (26)	1.18	0.277	A (52.3)
*CPEB3* rs11186856	AA (69)	AG (49)	GG (10)	0.10	0.753	A (73.0)
*SCN1A* rs10930201	AA (56)	AC (62)	CC (10)	1.62	0.203	A (68.0)
*SNAP25* rs3746544	GG (17)	GT (57)	TT (54)	0.10	0.750	G (35.5)
*WWC1* rs17070145	TT (20)	CT (59)	CC (49)	0.10	0.749	T (38.7)

## Data Availability

Data are available upon request due to restrictions (sensitive genetic and cognitive performance data of children and athletes).

## Supplementary Materials

The following Supporting Information can be downloaded at https://www.mdpi.com/article/10.3390/genes16050589/s1. Table S1: Cronbach’s α coefficients for the Kazakh and Russian versions of the Wechsler Intelligence Scales. Table S2: List of primers used for targeted next-generation sequencing. Table S3: Comparison of allele frequencies between study cohorts and control populations (Europeans and East Asians). Table S4: Comparison of tested variables between children and adults. Table S5: Summary of multiple regression models predicting self-reported foreign language proficiency.

## Abbreviations

The following abbreviations are used in this manuscript:

APOE	Apolipoprotein E
CAMTA1	Calmodulin-Binding Transcription Activator 1
CLSTN2	Calsyntenin 2
COMT	Catechol-O-Methyltransferase
CPEB3	Cytoplasmic Polyadenylation Element-Binding Protein 3
DNA	Deoxyribonucleic acid
EDTA	Ethylenediaminetetraacetic acid
IELTS	International English Language Testing System
IQ	Intelligence quotient
PCR	Polymerase chain reaction
SCN1A	Sodium Voltage-Gated Channel α Subunit 1
SNAP25	Synaptosome-Associated Protein 25
SNP	Single nucleotide polymorphism
TOEFL	Test of English as a Foreign Language
WAIS	Wechsler Adult Intelligence Scale
WISC	Wechsler Intelligence Scale for Children
WWC1	WW and C2 Domain-Containing 1
