# Supplementary material for: Association Between Genetically Predicted Memory and Self-Reported Foreign Language Proficiency
Source: Genes (Basel). 2025 May 17;16(5):589. doi: 10.3390/genes16050589 (PMC12111700; doi:10.3390/genes16050589)
Supplement: Supplementary file 1 [file genes-16-00589-s001.zip › genes-3586747-supplementary.pdf]

Association Between Genetically Predicted Memory and Self-Reported Foreign Language Proficiency

Yerdenova M.B. et al. 2025 (Genes)

Supplementary Materials

Supplementary Table S1. Cronbach's alpha coefficients for the Kazakh and Russian versions of the Wechsler Intelligence Scales.

**Tests**	**Language**	**Cronbach's alpha**
Wechsler Intelligence Scale for Children		
General	Kazakh	0.75
	Russian	0.82
Verbal	Kazakh	0.71
	Russian	0.77
Nonverbal	Kazakh	0.66
	Russian	0.68
Wechsler Adult Intelligence Scale		
General	Kazakh	0.83
	Russian	0.83
Verbal	Kazakh	0.78
	Russian	0.77
Nonverbal	Kazakh	0.67
	Russian	0.72

Supplementary Table S2. List of primers used for targeted next-generation sequencing.

Gene	Polymorphism	Forward Primer Sequence	Reverse Primer Sequence
CAMTA1	rs4908449	TTATTGGCCTATCTCCTTGCT	GAGAAAGATGGGCGGAGAG
CLSTN2	rs6439886	GGAAGAGGGGCAGAGATTG	TGAAACTGACAGTCGGCACA
COMT	rs4680	GAGATCAACCCCGACTGTG	CTGGTGGGGAGGACAAAGT
CPEB3	rs11186856	TGCTGTTTGACTTGGGTGGT	CTAAATTCAAGGATCAAGGGG
SCN1A	rs10930201	TGTTATCTACTTTCTGTTACTTG	CTTCTCTTGGCTAATTGTCTTA
SNAP25	rs3746544	ACACACATCAGTCCACCCC	AACAGCACATTGAGCATTCCT
WWC1	rs17070145	TACTCCCAGCACACACCTC	GTTGGCAGATGGAACCCGT

Supplementary Table S3. Comparison of allele frequencies between study cohorts and control populations (Europeans and East Asians).

**Polymorphism**	**Memory-increasing allele, %**					
	Allele	Kazakh children	East Asians*	Russian children	Slavic adults	Europeans*
CAMTA1 rs4908449	T	35.6	26.8	27.8	37.5	41.3
CLSTN2 rs6439886	G	6.8	7.4	11.1	13.3	11.8
COMT rs4680	A	19.8	28.0	30.6	52.3	50.0
CPEB3 rs11186856	A	91.0	86.6	86.1	73.0	68.0
SCN1A rs10930201	A	32.0	89.0	22.2	68.0	69.2
SNAP25 rs3746544	G	34.2	24.4	36.1	35.5	35.8
WWC1 rs17070145	T	60.4	76.5	47.2	38.7	35.4

* Data sourced from the Ensembl database (https://www.ensembl.org/Homo_sapiens/Info/Index)

Supplementary Table S4. Comparison of tested variables between children and adults.

**Variables, mean (SD)**	**Kazakh children (n=111)**	**Russian children (n=18)**	***p* value**	**Male children (n=66)**	**Female children (n=63)**	***p* value**
Age, years	14.4 (3.9)	13.4 (4.0)	0.3408	14.3 (3.8)	14.2 (4.0)	0.8401
Verbal IQ, points	121.1 (13.3)	131.7 (10.7)	0.0017*	121.4 (14.3)	123.7 (12.5)	0.3427
Self-reported foreign language proficiency, points	2.0 (0.9)	2.2 (0.9)	0.4537	1.9 (0.8)	2.1 (1.0)	0.2533
Level of immersion in foreign languages, points	0.45 (0.67)	0.78 (0.88)	0.0688	0.51 (0.64)	0.48 (0.77)	0.8541
Number of memory-increasing alleles (7 SNPs model)	5.6 (1.6)	5.2 (1.8)	0.371	5.4 (1.7)	5.7 (1.6)	0.2281
Number of memory-increasing alleles (5 SNPs model)	4.6 (1.3)	4.2 (1.0)	0.228	4.7 (1.3)	4.3 (1.3)	0.0574
**Variables, mean (SD)**	**Russian adults (n=107)**	**Belarusian and Ukrainian adults (n=21)**	***p* value**	**Male adults (n=90)**	**Female adults (n=38)**	***p* value**
Age, years	29.8 (8.3)	29.6 (7.7)	0.925	30.8 (8.2)	27.3 (7.6)	0.0282*
Self-reported foreign language proficiency, points	2.4 (0.9)	2.3 (1.0)	0.6112	2.4 (0.9)	2.6 (1.1)	0.2470
Number of memory-increasing alleles (7 SNPs model)	6.4 (2.0)	6.2 (1.6)	0.6511	6.4 (2.0)	6.3 (1.9)	0.7706
Number of memory-increasing alleles (6 SNPs model)	5.6 (1.8)	5.6 (1.7)	0.9958	5.6 (1.8)	5.6 (1.7)	0.9602

*$p < 0.05$, statistically significant differences between sub-groups, i.e. Kazakh children vs Russian children or male adults vs female adults (unpaired t test).

Supplementary Table S5. Summary of multiple regression models predicting self-reported foreign language proficiency.

**Cohort**	**Predictor**	**β (Beta)**	***p* value**
**Children**	**7 SNPs model**		
	Memory polygenic score (7 SNPs)	0.117	0.0078*
	Age	0.091	<0.0001*
	Verbal IQ	0.020	0.0004*
	Immersion in foreign languages	0.294	0.0035*
	Sex	0.863	0.3899
	Ethnicity	-0.001	0.9948
**Children**	**5 SNPs model**		
	Memory score (5 SNPs, HWE filter)	0.147	0.0091*
	Age	0.096	<0.0001*
	Verbal IQ	0.020	0.0003*
	Immersion in foreign languages	0.283	0.0048*
	Sex	0.097	0.4889
	Ethnicity	-0.019	0.9280
**Adults**	**7 SNPs model**		
	Memory polygenic score (7 SNPs)	0.103	0.0158*
	Age	0.014	0.1803
	Sex	-0.267	0.1492
	Ethnicity	-0.057	0.7986
**Adults**	**6 SNPs model**		
	Memory score (6 SNPs, HWE filter)	0.100	0.0371*
	Age	0.013	0.2162
	Sex	-0.252	0.1760
	Ethnicity	-0.081	0.7174

*$p < 0.05$, statistically significant predictor.
